# A Deep Dive into the Molecular and Immune Landscape of Undifferentiated Carcinomas with Osteoclast-like Giant Cells

**DOI:** 10.3390/cells15090837

**Published:** 2026-05-02

**Authors:** Eric Chang, Jiaqi Shi

**Affiliations:** Department of Pathology & Clinical Labs, University of Michigan, Ann Arbor, MI 48109, USA; ericpch@med.umich.edu

**Keywords:** pancreatic ductal adenocarcinoma, pancreatic cancer, mutations, tumor microenvironment, immunotherapy

## Abstract

**Highlights:**

**What are the main findings?**

- UCOGC is a distinct pancreatic carcinoma subtype that is genetically aligned with PDAC (recurrent KRAS, p53, CDKN2A, SMAD4 alterations) yet shows different clinicopathologic behavior, particularly improved outcomes in “pure” UCOGC.

**What are the implications of the main findings?**

- The distinct tumor microenvironments of UCOGC supports treating UCOGC as a PDAC-spectrum tumor with subtype risk stratification and should be evaluated for the use of PD-L1 immunotherapy and biomarkers.

**Abstract:**

Undifferentiated carcinoma with osteoclast-like giant cells (UCOGC) is a rare, distinct subtype of pancreatic carcinoma, formally classified separately from undifferentiated carcinoma (UC) of the pancreas in the World Health Organization’s 2010 and 2019 revisions. Whereas classic UC is associated with a poor prognosis and low survival rates, recent studies suggest that patients with UCOGC experience significantly longer survival and more frequent diagnosis at surgically resectable stages. Molecular profiling reveals that UCOGC consistently harbors canonical mutations in KRAS, CDKN2A, TP53, and SMAD4, aligning its classification within pancreatic ductal adenocarcinoma. In addition, UCOGC demonstrates a heterogeneous molecular landscape with distinctive mutations of uncertain biological relevance. Immunologically, UCOGC is characterized by a unique tumor microenvironment, notably a deficiency in regulatory T cells (Tregs) and a relative abundance of antigen-presenting cells. Elevated expression of PD-1 within UCOGC further suggests a potential for enhanced response to PD-1-targeted immunotherapies. Collectively, these findings underscore the need for ongoing research into the molecular and immunological characteristics of UCOGC, with the aim of identifying novel biomarkers and developing targeted treatment strategies.

## 1. Introduction

Pancreatic cancer is an exceptionally lethal malignancy that poses a significant global health challenge. Although it ranks as the 12th most common cancer worldwide, it is the sixth leading cause of cancer-related deaths [1]. In the United States, pancreatic cancer stands as the tenth most common cancer, accounting for 3.3% of new cancer diagnoses annually. Notably, it is the third leading cause of cancer mortality in the United States, reflecting its poor prognosis and high lethality. The 5-year survival rate remains strikingly low at just 13.3% [2]. The most prevalent form of pancreatic cancer is pancreatic ductal adenocarcinoma (PDAC), which is characterized by malignant glandular differentiation. Histologically, PDAC displays glands ranging from well- to poorly-formed, infiltrating the pancreatic parenchyma, often accompanied by luminal or intracellular mucin production [3].

Among the risk factors, tobacco smoking remains the most significant, although certain dietary factors have also been implicated in the development of pancreatic cancer. Clinically, more than half of all PDAC cases recur within 12 months following curative resection [4]. Survival rates vary markedly by stage: the overall 5-year survival rate across all stages of PDAC is approximately 13%, with patients diagnosed at a localized stage faring better (43.6%), compared to those with metastatic disease (3.2%) [2].

Molecular analyses across multiple studies have shown that PDAC exhibits recurrent genetic alterations, most notably in KRAS, TP53, CDKN2A, and SMAD4 [5,6,7,8]. Beyond establishing these core events, several groups have made foundational contributions to our molecular understanding of PDAC. Early work by Tuveson and Moffit et al. [6] helped characterize mutations and transcriptional programs spanning both tumor epithelium and stroma. Hruban and Yachida et al. [7] validated the stepwise molecular progression of PDAC and provided evidence that distant metastases arise from later-emerging subclones. Tuveson and colleagues also demonstrated that the precursor lesion pancreatic intraepithelial neoplasia (PanIN) frequently harbors KRAS mutations [9]. Expanding beyond the four canonical genes, Biankin et al. [8] identified additional recurrent alterations and implicated pathways involved in chromatin modification and DNA damage repair, helping to shape current models of PDAC molecular subtypes. Recently, Bailey et al. classified PDAC into squamous, pancreatic progenitor, aberrantly differentiated endocrine exocrine (ADEX), and immunogenic molecular subtypes [10]. PDAC also encompasses a broad range of histomorphologic variants, including adenosquamous carcinoma, squamous cell carcinoma, colloid carcinoma, hepatoid carcinoma, medullary carcinoma, micropapillary carcinoma, signet ring cell carcinoma, and undifferentiated carcinoma [3]. Additional rare patterns have been described, such as large duct [11], foamy gland [12], clear cell [13], and cystic papillary morphologies [14].

Among the rare histologic subtypes of PDAC, adenosquamous carcinoma is the most frequently observed, accounting for approximately 0.9–4.4% of exocrine pancreatic neoplasms [15]. By definition, adenosquamous carcinoma must contain at least 30% squamous carcinoma components in addition to conventional PDAC histology [3]. Prognostically, Boyd et al. analyzed 415 cases and found that adenosquamous carcinoma is associated with significantly worse two-year survival following surgical resection compared to PDAC (29% vs. 36%, *p* < 0.0001). Furthermore, adenosquamous carcinomas tend to be more frequently node-positive (52.8% vs. 47.1%, *p* < 0.0001), larger in size (5.7 cm vs. 4.3 cm, *p* < 0.0001), and more poorly differentiated (71.4% vs. 45.0%, *p* < 0.0001) [16]. Another rare variant is pancreatic squamous cell carcinoma, with an incidence of only 0.02 per 100,000 people, in stark contrast to PDAC’s incidence of 6.9 per 100,000. Squamous cell carcinoma of the pancreas is associated with particularly poor outcomes, as demonstrated by Makarova et al., who reported a one-year relative survival rate of 14% (95% CI: 9.5–19.4), compared to 24.5% (95% CI: 24.2–24.8) for PDAC [17].

Undifferentiated carcinomas (UC) of the pancreas exhibit a poorer prognosis compared to conventional PDAC and are further classified into three morphologic patterns: anaplastic undifferentiated carcinoma, sarcomatoid undifferentiated carcinoma, and carcinosarcoma. These UCs display limited differentiation, forming diffuse, sheet-like patterns lacking the glandular architecture typical of PDAC, and have an average survival of just 5 months [18,19].

Historically, undifferentiated carcinoma with osteoclast-like giant cells (UCOGC) was classified under UC. However, UCOGC is now recognized as a distinct entity, owing to its striking clinical and biological paradox. Despite often presenting as larger and significantly less differentiated than conventional PDAC, cases of UCOGC exhibit unexpectedly favorable outcomes compared to other poorly differentiated or undifferentiated pancreatic malignancies [20]. Notably, one study reported a 5-year survival rate of 59.1% for patients with UCOGC [18]. Nevertheless, the rarity of UCOGC leaves many important questions unanswered. First, the origin and function of the hallmark osteoclast-like giant cells remain unclear—whether these represent a mere immune reaction to carcinoma cells, or if they play tumor-promoting or tumor-limiting roles, is still under investigation. The osteoclast-like giant cells are shown to be histiocytic and positive for CD68 and lack cytokeratin staining. However, multiple studies debate whether they are ductal in origin with some studies showing KRAS mutations and having an epithelial-to-mesenchymal transition [21,22]. Although the role of these giant cells is unknown, the fact that pure UCOGC does better hints that they play a tumor-limiting role. Second, the tumor’s immune microenvironment diverges sharply from that of conventional PDAC, potentially opening new therapeutic avenues. PDAC is well studied to have a tumor microenvironment that limits the effectiveness of therapy due to the dense stroma. However, UCOGC has unique stroma with increased tumor-infiltrating lymphocytes, distinct immune microenvironment, and high PD-L1 expression leading to a tumor with relatively better outcomes. Yet, the reasons for this immune distinctiveness and its impact on tumor progression remains unknown. Third, the clinical behavior of UCOGC challenges traditional size-based staging systems, suggesting that future large-scale, multi-institutional studies are needed to better define its clinical characteristics and prognosis.

This paper aims to provide a comprehensive deep dive of UCOGC in hopes of answering these questions while also highlighting its distinguishing features compared to PDAC and UC. Additionally, the review will explore its tumor microenvironment, as well as emerging treatment options, such as those targeting PD-L1. Clarifying these mysteries of UCOGC could not only inform management of this rare subtype but also enhance our broader understanding of pancreatic cancer biology.

## 2. Classification

**Figure 1:**
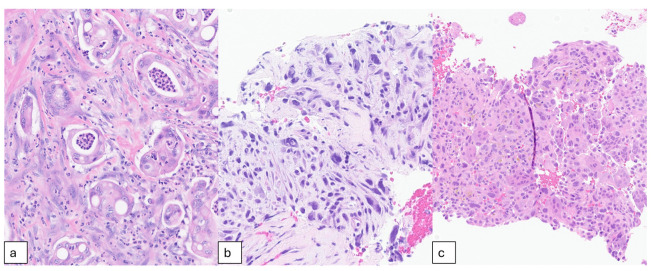
(**a**) Pancreatic ductal adenocarcinoma with moderately formed glands infiltrating desmoplastic pancreatic parenchyma. (**b**) Undifferentiated carcinoma of the pancreas with single pleomorphic epithelioid or spindled neoplastic cells without gland formation. (**c**) Undifferentiated carcinoma with osteoclast-like giant cells showing three cell types: osteoclast-like multinucleated giant cells, neoplastic mononuclear cells, and nonneoplastic mononuclear histiocytes. Images were obtained from the University of Michigan Pathology department.

Although the vast majority (85–90%) of pancreatic cancers are PDAC, UC of the pancreas is a rare but highly aggressive subtype. Reported frequencies vary widely, ranging from 0.4% to as high as 7% in earlier series [23,24]. More recent estimates suggest a substantially lower incidence, at approximately 0.4% of all pancreatic neoplasms [23]. Unlike PDAC, which is defined by glandular differentiation, UC is characterized by features such as poor cell cohesion, hypercellularity, and minimal stroma (Figure 1a,b) [3]. According to the World Health Organization, UC can be further subdivided into three morphologically distinct subtypes: anaplastic undifferentiated carcinomas, sarcomatoid undifferentiated carcinomas, and carcinosarcomas. It is important to note that undifferentiated carcinoma with osteoclast-like giant cells (UCOGC) is considered a separate entity from UC, as it exhibits distinct clinical behavior and outcomes [3].

UCOGC is an exceptionally rare variant of PDAC, accounting for approximately 0.4% of pancreatic carcinomas [20]. UCOGC was first described by Sommers and Meissner in 1954 as an “unusual carcinoma of the pancreas” [25]. Later In 1968, Juan Rosai referred to it as a “carcinoma simulating giant cell tumor of bone” [26]. Histologically, UCOGC is defined by three distinct cell populations: (1) neoplastic cytokeratin-positive mononuclear cells exhibiting nuclear atypia and pleomorphism, (2) non-neoplastic mononuclear histiocytes, and (3) non-neoplastic osteoclast-like multinucleated giant cells, typically found adjacent to areas of hemorrhage and necrosis (Figure 1c) [27].

## 3. Pathological Evaluation

A large meta-analysis by Imaoka revealed that PDAC most frequently arises in the head of the pancreas, representing 67.5% of all cases [28]. Similarly, Muraki’s study found an even higher prevalence in the head (83.7%) compared to the distal pancreas (16%, body/tail) [20]. In contrast, UC is less common in the pancreatic head, with only 43.4% of UC cases found in this location, and a slight predilection for the body and tail [28].

UCOGC can develop in any region of the pancreas, but multiple studies indicate a primary occurrence in the head. Christopher reported 48.7% of UCOGC cases in the pancreatic head [23], Lan found 36.2% in the head [29], and Muraki observed a higher proportion, with 61.1% located in the head [20]. These findings suggest a variable, but notable, tendency for UCOGC to arise in the pancreatic head, albeit less consistently than conventional PDAC. Importantly, studies have consistently shown that the anatomical site of UCOGC does not significantly impact overall survival rates, indicating that prognosis for UCOGC is independent of its primary location when compared to tumors lacking osteoclast-like giant cells.

Interestingly, UCOGC typically present at a larger mean size (5.3 cm) compared to conventional PDACs [20]. Paradoxically, despite their larger size, UCOGCs are associated with lower rates of lymph node metastasis and perineural invasion (PNI). The study by Muraki demonstrated that only 22.6% (7/38) of UCOGC cases had lymph node metastasis compared to 64% (461/725) of conventional PDAC cases (*p* < 0.0001). As expected, negative lymph node status is independently associated with improved survival in patients with UGOGC, while lymph node positivity correlates with poorer outcomes. Interestingly, the rate of lymphovascular invasion (LVI) was found to be similar between UCOGC and PDAC, with Muraki reporting LVI in 62.9% of UCOGC cases and 62.2% of PDAC cases [20]. In contrast, UCOGCs exhibited consistently lower rates of PNI, with only 31.6% of cases demonstrating PNI, compared to 85.5% in PDAC [20].

Extrapolating from both the presence of positive lymph nodes and perineural invasion, UCOGC appear to be less invasive despite its larger tumor size than PDAC [20]. This clinical-pathological paradox is currently not explained by literature. Critically, this pattern contradicts the usual expectation that larger tumors are more locally and regionally invasive. One hypothesis is that the robust immune infiltrate observed in UCOGC could exert a mechanical or immunological restraint on lymphatic and perineural invasions.

Muraki also examined the surgical margin status comparing PDAC to UCOGC. PDAC achieved a negative surgical margin rate of 75.5% (545/ 725), whereas UCOGC demonstrated a higher negative margin rate of 87.1% (27/31). However, this difference was not statistically significant (*p* = 0.1385) [20]. A review by Christopher reported a lower rate of negative surgical margins in UCOGC. Importantly, the presence of a positive margin was associated with reduced overall survival (*p* = 0.002), highlighting the importance of achieving complete resection in patients with UCOGC [23]. These differences in negative resection margins could be explained by the lower rates in perineural invasion seen in UCOGC.

In Muraki’s study, 76% of UCOGC cases exhibited an invasive ductal adenocarcinoma component, which comprised anywhere from less than 5% to as much as 80% of the tumor, with a mean proportion of 21%. Additionally, four cases (11%) of UCOGC arose within a mucinous cystic neoplasm (MCN), and another four cases (11%) were associated with an intraductal papillary mucinous neoplasm (IPMN). Notably, all tumors associated with MCN and IPMN demonstrated areas of high-grade dysplasia [20].

Table 1 provides a comparative overview of the histological, molecular, and tumor microenvironment characteristics of UCOGC, PDAC, adenosquamous carcinoma, and UC.

## 4. Prognosis

Initially, UCOGC was widely perceived as a highly aggressive malignancy, with an impression that its prognosis was similar to, or even worse than, that of conventional PDAC. Earlier studies often reported mean survival times of UCOGC as low as 12 months. However, this unfavorable prognosis is now considered somewhat misleading, as much of the early literature grouped it together with other undifferentiated or anaplastic pancreatic carcinomas, obscuring its true clinical course [20].

Recent research that distinguishes UCOGC from other neoplasms has revealed important prognostic differences. This distinction is typically defined in terms of “pure” versus “non-pure” UCOGC. Pure UCOGC comprises tumors exclusively displaying the characteristic UCOGC morphology—namely, neoplastic and non-neoplastic mononuclear cells and osteoclast-like giant cells—without any associated component of another pancreatic neoplasm. In contrast, non-pure UCOGC refers to tumors where UCOGC morphology coexists with other forms of pancreatic neoplasms such as PDAC, MCN, or IPMN [30].

Several studies underscore the impact of this distinction on prognosis. For example, Luchini et al. demonstrated significant differences in survival based on UCOGC purity. In their cohort of 22 UCOGC cases, patients with pure UCOGC had a notably better prognosis, with a median survival of 36 months (IQR: 16–72 months), compared to those with UCOGC associated with adenocarcinoma, who had a median survival of 15 months (IQR: 9–32 months; log-rank test: *p* = 0.04). After adjusting for age and sex, the study found that patients with UCOGC associated with conventional PDAC had nearly a five-fold higher risk of mortality (Hazard Ratio [HR] = 4.98; 95% CI: 1.00–24.82; *p* = 0.05) [30].

Additionally, Mills et al. reported significantly longer median overall survival for patients with UCOGC compared to those with UC across all stages (10.8 years versus 0.4 years, *p* = 0.003) [31]. Among resected cases, median survival for UCOGC was 11.9 years, versus 1.8 years for UC, although this difference did not reach statistical significance (*p* = 0.08) [31].

That said, other studies have yielded more nuanced results. In a clinicopathological analysis of 38 resected UCOGCs, Muraki et al. found that the 5-year survival rate was 100% for the 9 cases lacking a PDAC component. In contrast, the 16 cases with limited PDAC components (<10% of the tumor) and those with focal PDAC components (10–50% of the tumor) had 5-year survival rates of 50% and 80%, respectively. No calculable survival rate was available for tumors with more than 50% PDAC component. Despite these apparent trends, Muraki et al. reported that median survival across these groups was not significantly different (*p* = 0.5385), nor was there a significant difference between pure UCOGC and all other cases (*p* = 0.6394). It is important to note that, of the 38 cases examined in Muraki’s study, only 9 were classified as pure UCOGC, potentially resulting in insufficient statistical power [20]. Although both the Muraki and Luchini studies demonstrated a general trend toward better prognosis in cases with a 100% pure UCOGC phenotype, Muraki’s findings did not reach statistical significance. A possible explanation is that, unlike Luchini, Muraki’s cohort included patients who received adjuvant chemotherapy following surgery, which could substantially affect survival outcomes and account for the lack of significant difference based on UCOGC purity.

## 5. Clinical Management

Due to the rarity of these pancreatic cancer subtypes, there are currently no standardized treatment guidelines for UCOGC. Management strategies are typically extrapolated from small reviews, case reports, and established practices for more common pancreatic cancers. At present, the only potentially curative option for UCOGC is surgical resection [23]. Mills et al. demonstrated that achieving a microscopically negative surgical resection margin (R0 resection) is independently associated with improved overall survival; patients with resected UCOGC had a median overall survival of 11.9 years compared to only 1.8 years for those with resected UC [31]. Remarkably, some studies report R0 resection rates as high as 100% for UCOGC, highlighting the potential for excellent surgical outcomes in this patient population [31].

Beyond surgery, chemotherapy regimens used for PDAC have been adopted for UCOGC, although their efficacy remains uncertain due to limited data. While adjuvant chemotherapy using agents such as gemcitabine or FOLFIRINOX has no significant improvements in survival outcomes in some studies [32,33], other reports recommend its use to potentially reduce recurrence and enhance long-term survival when using gemcitabine plus nab-paclitaxel [32]. The evidence for neoadjuvant chemotherapy is similarly mixed. Some advocate for its use to downstage locally advanced or borderline resectable UCOGC in order to facilitate R0 resection, while others note that the current evidence is insufficient to draw firm conclusions.

Surgical resection remains the only potentially curative treatment for UCOGC, and its technical aspects are noteworthy given the unique pathology of this subtype. Negative margin (R0) resections are achieved more frequently in UCOGC (87.1%) than in conventional PDAC (75.5%), although this difference does not always reach statistical significance. The comparatively lower rates of lymph node metastasis and PNI in UCOGC contribute to greater resectability, despite the larger average size at presentation. While UCOGC does not require unique surgical techniques, its characteristic “pushing borders” and lack of infiltrative growth often enable safer and more complete resections, sometimes reducing operative complexity. The increased R0 resection rate in UCOGC underscores the potential for curative outcomes with surgery. Nevertheless, surgeons should be vigilant for the rare coexistence of other neoplastic components (e.g., associated PDAC or IPMN), as these may alter disease aggressiveness and necessitate changes in operative strategy and adjuvant therapy.

## 6. Molecular Features

Historically, the molecular landscape of PDAC has been shaped by several pivotal studies. Using human and murine models, Tuveson showed that PanIN lesions frequently harbor early KRAS mutations, followed by loss of CDKN2A (p16). TP53 and SMAD4 alterations were observed less commonly and tended to appear in higher-grade PanINs. The subsequent development of genetically engineered mouse models recapitulating PanIN formation and progression to invasive carcinoma provided key experimental validation of this stepwise molecular evolution toward PDAC [9]. Moffitt and Tuveson were also among the first to deconvolute tumor and stromal expression programs by separately profiling microdissected epithelial and stromal compartments. This work described classical and basal-like tumor subtypes, along with normal and activated stromal subtypes, foreshadowing contemporary PDAC subtype frameworks. In parallel, Yachida and Hruban et al. demonstrated that the capacity for distant metastasis is typically acquired late, arising from advanced subclones. Metastases shared many alterations with the primary tumor, with additional “branch” mutations emerging during subclonal evolution--helping explain PDAC’s often rapid and aggressive clinical course [7]. Extending beyond the four canonical driver genes, Biankin et al. identified 16 significantly mutated genes, implicating additional pathways such as chromatin modification (e.g., EPC1, ARID2), DNA damage repair (e.g., ATM), and axon guidance (e.g., SLIT2, ROBO2) [8]. This pathway-centric perspective helped motivate subsequent studies focused on therapeutically relevant molecular circuits. Finally, Scarpa played an important role in defining the molecular landscape of pancreatic neuroendocrine tumors (PNETs), highlighting recurrent alterations involving DNA damage repair (MUTYH, CHEK2, BRCA2), chromatin remodeling (MEN1, SETD2, ARID1A, MLL3), telomere maintenance (TERT), and mTOR pathway dysregulation (PTEN, TSC2, TSC1, DEPDC5) [34].

In the landmark study by Bailey et al., whole genome sequencing was performed on 456 cases of PDAC and its variants, including adenosquamous carcinoma, colloid carcinoma, and PDAC associated with intraductal papillary mucinous neoplasm (IPMN); a small number of acinar cell carcinomas were also analyzed. This comprehensive genomic analysis identified four common driver mutations—KRAS, TP53, SMAD4, and CDKN2A-alongside a wide array of other low prevalence gene alterations. Despite the mutation heterogeneity, these oncogenic mutations were found to converge into four molecular subtypes, each characterized by dysregulation of core pathways such as DNA damage repair, cell cycle regulation, TGF-beta signaling, chromatin remodeling, and axonal guidance. The four subtypes described by Bailey et al. are: squamous, pancreatic progenitor, immunogenic, and aberrantly differentiated endocrine exocrine (ADEX) [10].

The squamous subtype is notable for enrichment of TP53 mutations and distinctive alterations such as KDM6A inactivation (a chromatin remodeling gene) and TP63∆N (a p53 family member). Cooperation between TP63∆N and TP53 mutations was implicated in driving epithelial-to-mesenchymal transition [10,35]. This subtype also exhibited hypermethylation of pancreatic endodermal cell fate determinant genes, including PDX1, GATA6, and HNF1B.

The pancreatic progenitor subtype is defined by mutations in transcription factors critical for pancreatic differentiation: PDX1, MNX1, HNF4G, HNF1B, HNF1A, FOXA2, FOXA3, and HES1. Notably, PDX1 plays an essential role in embryonic pancreatic progenitor cells. This subtype is further characterized by genes regulating fatty acid oxidation, steroid hormone synthesis, and mucin glycosylation, with preferential expression of MUC5AC and MUC1—markers of the pancreatobiliary subtype of IPMN and histologic PDAC-associated IPMN.

The ADEX subtype comprises tumors with transcriptional signatures for later pancreatic development, spanning both exocrine and endocrine differentiation. This group is considered a subclass of pancreatic progenitor tumors, with key transcription factors including NR5A2, MIST1, and RBPJL for exocrine differentiation, and INS, NEUROD1, NKX2-2, and MAFA for endocrine differentiation. Additional genes associated with terminally differentiated pancreatic tissue, such as AMY2B, PRSS1, PRSS3, CEL, and INS, were also identified.

Finally, the immunogenic subtype is distinguished by abundant immune cell infiltration, including B cell signaling, antigen presentation, CD4+ and CD8+ T cell signaling, and Toll-like receptor pathways. The immune infiltrate is predominately composed of cytotoxic (CD8+) and regulatory (CD4+) T cells.

Multiple studies, including those by Mills et al., have consistently identified molecular alterations in UCOGC that are also present in PDAC, reinforcing the classification of UCOGC as a subtype of PDAC [31,36,37]. The four most commonly shared mutations in both entities are KRAS, TP53, CDKN2A, and SMAD4. UCOGC has also been reported to harbor less frequent mutations, such as SERPINA3, GLI3, MAGEB4, MEGF8, TTN, BAP1, PTEN, BRCA2, BRAF, and IDH [30]. In the largest cohort to date, Hrudka et al. used next-generation sequencing on 13 UCOGC cases and identified additional pathogenic or likely pathogenic mutations commonly found in PDAC, including CIC, GNAS, APC, ATM, NF1, FBXW7, ATR and FGFR4 [27]. Zhao et al. further nominated additional possible gene mutations such as AR, CCNE1, and BTK [38]. While the four primary mutations (KRAS, TP53, CDKN2A, and SMAD4) are central to the molecular profile of PDAC and UCOGC, the numerous less frequent mutations identified thus far have no significant association with pancreatic cancer outcomes or pathogenesis.

KRAS plays a pivotal role in the development and progression of PDAC and its variants, including UC and UCOGC. Asa member of the Ras family of small GTPases, KRAS is central to intracellular signaling, activating multiple downstream pathways that drive oncogenic transformation. Notably, Ras signaling influences key cascades such as MAP kinase, PI3K/AKT/mTOR, and phospholipase C [39,40,41]. KRAS mutations, highly prevalent in PDAC-detected in approximately 85% to 93% of cases according to various studies [27]—are most frequently activating mutations at codon 12 (e.g., p.Gly12Asp, p.Gly12Val, p.Gly12Arg). These mutations also occur in other malignancies, present in roughly 45% of colorectal adenocarcinomas, 30% of lung adenocarcinomas, and at lower frequencies in additional tumor types. In UCOGC, KRAS mutations are similarly common; one study found all eight UCOGC cases harbored KRAS mutations [30], while another found mutations in ten out of thirteen cases [27]. Importantly, the non-neoplastic osteoclast-like giant cells in UCOGC lack KRAS mutations, while the neoplastic pleomorphic mononuclear cells possess them, supporting a shared ductal epithelial origin [21]. Given the centrality of KRAS-driven pathways in PDAC and its variants, combined therapeutic strategies targeting Ras effectors—such as MEK/ERK inhibitors with immunotherapy-warrant further investigation [42].

TP53 is a crucial tumor suppressor gene responsible for maintaining cell cycle regulation and facilitating programmed cell death (apoptosis). When TP53 is mutated, its ability to activate genes involved in cell cycle arrest and apoptosis is compromised, leading to unchecked cellular proliferation. In PanIN lesion, TP53 mutations commonly arise after the loss of CDKN2A, particularly in advanced lesions [43]. TP53 is recognized as one of the four seminal driver genes in PDAC, with inactivating mutations frequently marking the transition from PanIN to invasive carcinoma. A pivotal study by Kanda et al. demonstrated the presence of mutant TP53 alleles in pancreatic juice obtained from patients with PanIN displaying high-grade dysplasia. This finding underscores the potential of TP53 mutation analysis in pancreatic juice as a promising approach for early detection of pancreatic neoplasia [44]. The prevalence and pattern of TP53 mutations are consistent across PDAC and its variants, including UC and UCOGC. Next-generation sequencing has revealed TP53 mutations in 74% of PDAC cases [27]. Correspondingly, two independent studies of UCOGC reported TP53 mutations in 7 out of 8 cases and in 9 out of 13 cases, respectively [27,30]. Further, Muraki et al. used immunohistochemical analysis to show that non-neoplastic osteoclast-like giant cells are negative for p53, while pleomorphic mononuclear neoplastic cells and histocyte-like carcinoma cells demonstrate frequent p53 positivity [20].

CDKN2A, also known as p16 or p16INK4, is another pivotal tumor suppressor gene involved in pancreatic cancer pathogenesis. The intact CDKN2A gene encodes the p16 protein, which serves as a key regulator of cell cycle progression at the G1/S checkpoint. p16 functions by inhibiting cyclin-dependent kinases 4 and 6 (CDK4/6), thereby preventing phosphorylation of the retinoblastoma (Rb) protein and restraining uncontrolled cellular proliferation [43,45]. Whole-genome sequencing data reported by Hrudka indicate that 35% of PDAC cases exhibit alterations in CDKN2A. Research has also demonstrated that UCOGC shares a similar mutation profile with PDAC regarding CDKN2A, with mutations identified in 4 out of 13 UCOGC cases. Notably, loss of CDKN2A function in UCOGC may result from homozygous deletions, as confirmed using fluorescence in situ hybridization analysis. These findings further support a ductal epithelial origin for UCOGC [27].

SMAD4 (small mothers against decapentaplegic 4) is another major tumor suppressor gene implicated in PDAC. Inactivating mutations of SMAD4 promote progression to invasive carcinoma, with studies reporting SMAD4 mutations in 31% [27] and up to 55% [46] of PDAC cases. In contrast to other commonly mutated genes, SMAD4 alterations are observed less frequently in UCOGC. Whole genome sequencing studies have reported low rates of SMAD4 mutations, ranging from one out of thirteen cases (7.7%) in Hrudka’s study to one out of eight cases (12.5%) in Luchini’s study. However, immunohistochemical studies have unexpectedly shown loss of SMAD4 protein expression in approximately half of UCOGC cases (9/19, 47.4%) [27,30]. This discrepancy may be explained by differences in the detection methods: genomic sequencing identifies only those cases where the SMAD4 gene is mutated or deleted, while immunohistochemistry reveals a loss of protein expression, which can occur through other mechanisms such as epigenetic silencing, post-transcriptional regulation, or protein degradation even in the absence of a genetic mutation. Thus, non-mutational regulatory factors may contribute to the higher rate of SMAD4 loss detected by immunohistochemistry. Notably, the SMAD4 mutations identified in UCOGC closely resemble those found in conventional PDAC, further supporting the classification of UCOGC as a subtype within the PDAC spectrum [27].

While most UCOGCs display the classic mutations commonly observed in PDAC, recent studies have reported a heterogenous array of rare genetic alterations that occur considerably less frequently [27,30,31]. Although the presence of these mutations is noteworthy, caution is warranted in interpreting their significance as most findings are limited to small cohorts and their functional roles in UCOGC remain speculative or unknown. Of particular interest, Luchini et al. identified recurrent pathogenic mutations in SERPINA3 and GLI3 across multiple UCOGC cases, whereas other rare mutations were observed only in single cases [30]. SERPINA3 encodes alpha 1-antichymotrypsin, a member of the serine protease inhibitor (serpin) family. As an acute-phase reactant secreted by hepatocytes, alpha 1-antichymotrypsin plays an important role in modulating the anti-inflammatory response [47]. SERPINA3 has been studied in a variety of malignancies, including squamous cell carcinoma of the esophagus [48], melanoma [49], and endometrial cancer [50], where its mutations are frequently associated with aggressive disease and poor prognosis in colon, lung, and gastric cancers. Although these clinical associations suggest a role in cancer pathobiology, the specific mechanisms by which SERPINA3 mutations drive tumorigenesis are not fully understood. It is hypothesized that SERPINA3 may influence cancer progression by regulating the transcription of key oncogenes [47]. In the study by Luchini et al., two out of 22 UCOGC cases harbored a SERPINA3 driver mutation [30]. To date, only one study conducted by Mawaribuchi has specifically investigated SERPINA3 as a glycobiomarker in PDAC, revealing a lack of SERPINA3 expression in normal pancreatic tissue. Notably, there are currently no additional reports documenting SERPINA3 mutations in UC [51].

GLI3 is another notable mutation that may help distinguish UCGOC from other forms of pancreatic cancer. GLI3 encodes a transcription factor central to the Hedgehog signaling pathway, which is critically involved in pancreatic tumorigenesis and is upregulated in various malignancies [52]. Research indicates that GLI3 can promote malignant cellular behavior, with mutations identified in gastric cancer [53], PDAC [54], and glioblastoma [55]. In the study by Luchini et al., GLI3 mutations were detected in two UCOGC cases, suggesting a potential role in the tumorigenesis of this variant [30]. Despite these findings, research on GLI3 in pancreatic cancers remains limited, particularly regarding its function in UC and UCOGC. To date, no studies have identified GLI3 mutations in UC alone. It is important to note that both SERPINA3 and GLI3 are relatively under-investigated and currently lack robust clinical significance. Additional studies with larger cohorts are needed to elucidate any meaningful molecular distinctions between UCOGC, UC, and PDAC beyond the established four main driver mutations.

MicroRNAs (miRNAs) are small, non-coding RNAs that regulate gene expression at the post-transcriptional level and play crucial roles in cancer progression. They represent promising targets for therapeutic intervention. Several miRNAs-such as miR-21, miR-210, miR-155, and miR-196a have been implicated in the pathogenesis of PDAC [56]. In addition to genomic sequencing, Popov et al. analyzed microRNA profiles in UCOGC, recognizing the pivotal regulatory functions of miRNAs in both benign and malignant cells. Their study demonstrated that miRNA expression patterns in UCOGC closely resembled those observed in poorly differentiated PDAC, with no significant differences identified [57].

## 7. Tumor Microenvironment and Its Implication in Immunotherapy

Tuveson’s research has demonstrated that the pancreatic tumor microenvironment (TME) profoundly influences the tumor’s response to chemotherapy, cytotoxic therapies, and immunotherapy. In PDAC, Tuveson and Kleef et al. found that the tumor stroma is predominant and highly heterogenous, comprising pancreatic stellate fibroblast cells, diverse immune cell populations, and various extracellular matrix (ECM) components [58]. Mouse co-culture models of PDAC and pancreatic stellate cells revealed increased tumor proliferation, size, and metastasis frequency, with implicated mechanisms involving MAP kinase, PDGF, and TGFβ signaling pathways. The ECM-consisting of collagen, fibronectin, proteoglycans, and hyaluronic acid-compresses blood vessels, impeding drug delivery. Supporting this, Olive demonstrated that the active metabolite of gemcitabine was significantly higher in stroma-poor xenografts and nearly undetectable in stroma-rich tumors, further corroborating the barrier effect of dense stroma [59]. Chronic inflammation is a risk factor for PDAC development, with immune cells comprising roughly 50% of the tumor mass in Tuveson’s study. Notably, regulatory T-cells predominate over cytotoxic T-cells in the immune infiltrate [18,19,23,26,29,54,55,56,60].

While the TME is well-characterized in PDAC, there is comparatively limited literature regarding UCOGC. Immunotherapy, particularly through programmed death-ligand 1 (PD-L1) blockade, has emerged as a promising treatment for UCOGC. Tumors of this subtype frequently exhibit high PD-L1 expression-significantly more than conventional PDAC [61]. Studies have reported PD-L1 expression in about 60–80% of UCOGC cases, compared to just 12.5–16% in PDAC. Through immunohistochemistry, Hrudka specifically found PD-L1 expression in 76.9% of UCOGC cases versus only 12.5% of PDAC (*p* < 0.001) [61]. Importantly, elevated PD-L1 expression in UCOGC is associated with poorer prognosis. Although the precise mechanism is still unclear, it is hypothesized that PD-L1’s immunosuppressive effects enable tumor cells to evade cytotoxic T cell activity, contributing to aggressive behavior. Supporting this, UCOGC tumors harbor a unique immune microenvironment with a statistically significant increase in tumor-infiltrating lymphocytes when compared to conventional PDAC [34,61]. This distinctive immune landscape may explain the relatively better outcomes and less aggressive local invasion seen in UCOGC patients.

Further analysis of immune cell populations in UCOGC tumors indicates an increase in antigen-presenting cells such as M2 macrophages and natural kill cells, coupled with a decrease in cytotoxic T-cells and regulatory T-cells (Tregs). This composition suggests that the UCOGC microenvironment may be particularly receptive to immune-modulating therapies [31] Notably, this profile differs from that of PDAC, in which regulatory T-cells are the predominant immune cell population.

Clinical evidence for immunotherapy efficacy is mounting: Pembrolizumab, a PD-1 blocking monoclonal antibody, has shown effectiveness in metastatic UCOGC across several studies [62,63,64]. For example, Besaw et al. described a patient with metastatic pancreatic UCOGC who underwent 46 cycles of pembrolizumab, achieving complete elimination of liver and brain metastases and significant reduction of the primary pancreatic tumor [64]. Similarly, Obayashi et al. reported complete remission of lung metastasis after 8 months of pembrolizumab treatment [65]. Intriguingly, PD-1 inhibitors appear to have a more pronounced effect on metastatic disease than on the primary tumor, possibly due to differences in TME, such as increased tumor-infiltrating lymphocytes and higher PD-L1 expression in metastatic sites. These observations support routine PD-L1 immunohistochemistry testing in all metastatic UCOGC cases to inform immunotherapy decisions. However, it is important to note the absence of clinical trials specifically investigating pembrolizumab in UCOGC patients.

Although numerous molecular studies have explored pancreatic cancer, relatively few have investigated the overall tumor microenvironment and its potential influence on immunotherapy responsiveness. Mills et al. examined UC and UCOGC, comparing their cellular composition to that of conventional PDAC using both bulk RNA sequencing and multiplex immunofluorescence [31]. Their findings revealed that UC and UCOGC tumors are enriched in activated natural killer cells, activated mast cells, plasma cells, follicular helper T cells, gamma-delta T cells, and M2 macrophages. In contrast, PDAC samples exhibited higher levels of regulatory T-cells, as well as predominance of resting natural killer cells and naïve B cells. Notably, cytotoxic T lymphocytes were significantly less frequent in UC and UCOGC compared to PDAC. Additionally, both UC and UCOGC demonstrated lower abundance of regulatory T-cells relative to PDAC. UCOGCs were distinguished from PDAC by a significant enrichment of antigen-presenting cells expressing CD163. Assessment of Arginase-1-a marker associated with immunosuppressive tumor-associated macrophages-showed a trend toward decreased immunosuppressive macrophages in UCOGC compared to UC. However, investigation of individual osteoclast-like giant cells did not reveal specific immunological patterns [31].

**Figure 2:**
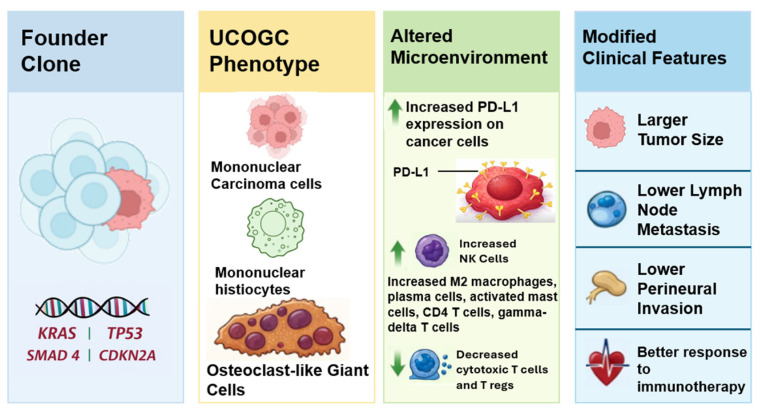
Schematic model of proposed UCOGC pathogenesis. A founder clone undergoes distinct stromal reprograming, giving rise to the UCOGC phenotype characterized by three hallmark cell types, an altered tumor microenvironment, and modified clinical features.

The observed enrichment of activated natural killer cells and mast cells, alongside a decrease in regulatory T-cells, may underlie the reduced aggressiveness of UCOGC relative to PDAC. It is hypothesized that differences in prognosis between UCOGC and PDAC could stem from distinct immune microenvironments, with UCOGC potentially being more responsive to immune-modulating therapies While these findings shed valuable light on the unique tumor microenvironment of UCOGC, further research is essential to comprehensively characterize this rare tumor type. Additional studies with larger cohorts are needed to validate these initial observations and to clarify significance of microenvironmental differences between UCOGC and PDAC. A summary figure was provided to illustrate the proposed UCOGC pathogenesis (Figure 2).

Another promising therapeutic option is allogenic NKG2D chimeric antigen receptor (CAR)-T cell therapy. CAR-T has been used with clinical success in various hematologic malignancies including B-cell acute lymphoblastic leukemia and large B-cell lymphoma. However, its use in solid tumors have been less established and with relatively limited success compared to its hematologic counterpart. Part of this is due to the tumor microenvironment in solid tumors inhibiting the CAR-T cells [66]. However, early preclinical studies of pancreatic and ovarian cancers show possible therapeutic use [67]. Farooq et al. demonstrated in mouse models of pancreatic cancers that tumor necrosis factor-α-induced protein 8-like 2 (TIPE2) deficient CAR-T therapy was more effective than conventional CAR-T. TIPE2 is an immune checkpoint protein known to suppress T-cell activation and effector function. His study demonstrated that in TIPE2 deficient CAR-T cells, the tumors had higher expression of activation, degranulation, and cytotoxic markers which resulted in more efficient tumor cell elimination compared to conventional CAR-T cells. Interestingly, this same concept could be applied to UCOGC as they contain less regulatory T-cells and have an increase in activated NK cells. These features might hint that CAR-T therapy might be a potential therapeutic avenue with good promise [68].

## 8. Conclusions

Pancreatic cancer remains one of the most formidable challenges in oncology, characterized by persistently high mortality rates. Among its various histopathological subtypes, UCOGC is particularly notable for its distinctive clinical, morphological, and molecular features. Despite often presenting with larger tumors, UCOGC tends to display less invasive behavior and is associated, especially in its pure form, with a relatively favorable prognosis compared to conventional PDAC and UC. Emerging evidence suggests that differences in the immune microenvironment play a pivotal role in shaping the clinical trajectory of UCOGC. Despite these studies, there are multiple unmet needs regarding treatment of UCOGC. There is an absence of subtype-specific treatment guidelines with limited evidence on when to use adjuvant/neoadjuvant chemotherapy. In addition, there are no validated biomarkers stratifying pure versus mixed UCOGC.

Genetic analyses consistently demonstrate that UCOGC shares the principal molecular drivers with PDAC, including mutations in KRAS, TP53, CDKN2A, and SMAD4, reinforcing its classification as a variant of PDAC rather than a distinct pathological entity. Notably, the relatively high expression of PD-L1 and the presence of unique immune cell populations in UCOGC further underscore its potential sensitivity to immunotherapeutic interventions. However, robust clinical data on immunotherapy in UCOGC remain limited, highlighting a clear need for further research in this area. Immunotherapy has shown to be useful due to its modulatory effect on the enriched anti-tumor immune cells’ microenvironment and high expression of PD-L1. T-cell exhaustion is an important characteristic of tumors, as cancers with an exhaustion phenotype are associated with poor prognosis. Markers specific for exhaustion have not yet been studied in the context of UCOGC, highlighting a gap in our understanding. Possible markers include chemokines, cytokines, and inhibitory receptors, such as CTLA-4, PD-1, TIM-3, LAG-3, and ITIM [69].

The management of UCOGC is hampered by its rarity, the absence of standardized treatment guidelines, and a reliance on therapeutic strategies extrapolated from more prevalent forms of pancreatic cancer. Surgical resection continues to represent the cornerstone of potentially curative therapy. The roles of adjuvant and neoadjuvant chemotherapy, as well as novel immunotherapeutic approaches, are subjects of ongoing investigation. To enhance our understanding and improve outcomes for patients with these uncommon pancreatic cancer subtypes, further multi-institutional studies and comprehensive molecular profiling are essential. Such efforts will be key in clarifying the prognostic and therapeutic implications unique to UCOGC and may help pave the way for more tailored clinical management in the future.

## Figures and Tables

**Table 1:** Comparison of histological, molecular, and tumor microenvironment characteristics among UCOGC, UC, adenosquamous carcinoma, and PDAC.

Pancreatic Cancer Subtypes	Histology	Molecular Findings	Tumor Microenvironment
Pancreatic ductal adenocarcinoma	Well- to poorly-formed glands haphazardly infiltrate desmoplastic stroma. Neoplastic cells are cuboidal or columnar with eosinophilic or mucinous cytoplasm and pleomorphic nuclei.	- **KRAS:** Mutated in 85% of cases - **TP53:** Mutated in ~75% - **CDKN2A/p16:** Loss or mutation common - **SMAD4/DPC4:** Lost in ~55% - **Other:** NOTCH, BRCA2, ARID1A, RNF43 mutations	- Dense desmoplastic stroma with activated fibroblasts (CAFs) - Immunosuppressive: High Tregs, MDSCs, M2 macrophages - Poorly infiltrated by cytotoxic T cells - Hypoxic, fibrotic microenvironment
Adenosquamous carcinoma	Mixture of glandular (adenocarcinoma) and squamous components; enlarged nuclei; keratinization; necrosis	KRAS, TP53, CDKN2A, SMAD4 mutations (like adenocarcinoma); EGFR overexpression possible	Prominent desmoplastic stroma; higher inflammatory infiltration; aggressive, immunosuppressive milieu.
Undifferentiated carcinoma	No definitive epithelial or glandular differentiation. Diffuse sheet-like growth pattern that is poorly cohesive and hypercellular. Stroma often is scant.	Most harbor KRAS and TP53 mutations similar to PDAC - **SMAD4:** More frequently retained compared to PDAC [27,30] - Loss of epithelial differentiation markers	- Highly aggressive - Frequently shows increased inflammatory cell infiltration - Less pronounced desmoplastic stroma than PDAC - Variable immune cell content
Undifferentiated carcinoma with osteoclast-like giant cells	Contains 3 distinct cell types: non-neoplastic osteoclast-like multinucleated giant cells, mononuclear histiocytic cells, and neoplastic mononuclear cells. The neoplastic cells display marked pleomorphism and a lack of cohesion, ranging in morphology from spindle-shaped to epithelioid forms.	- **KRAS:** Commonly mutated (similar to PDAC) - **TP53:** Mutated in 69.2%~87.5% [27,30] - **SMAD4:** up to 12.5% mutated by sequencing, 47.4% protein loss by IHC - Overexpression of EMT markers (vimentin), loss of E-cadherin - Unique gene expression profile relative to PDAC	- Osteoclast-like giant cells: CD68+ (macrophage phenotype), non-neoplastic - Rich in inflammatory cells (T cells, histiocytes) - Less desmoplastic stroma than PDAC - Prominent immune response

## Data Availability

No new data were created or analyzed in this study.
